# The Role of Lead and Cadmium in Gynecological Malignancies

**DOI:** 10.3390/antiox11122468

**Published:** 2022-12-15

**Authors:** Gabriela Furtak, Mateusz Kozłowski, Sebastian Kwiatkowski, Aneta Cymbaluk-Płoska

**Affiliations:** 1 Department of Reconstructive Surgery and Gynecological Oncology, Pomeranian Medical University in Szczecin, Al. Powstańców Wielkopolskich 72, 70-111 Szczecin, Poland; 2 Department of Obstetrics and Gynecology, Pomeranian Medical University in Szczecin, Al. Powstańców Wielkopolskich 72, 70-111 Szczecin, Poland

**Keywords:** lead, cadmium, metalloestrogen, gynaecological, endometrial cancer, ovarian cancer, cervical cancer

## Abstract

Lead and cadmium are non-essential and toxic heavy metals. Their presence and elevated levels can lead to many pathologies. They disrupt the antioxidant properties of many enzymes, consume the resources of antioxidant cells, and thus participate in the generation of oxidative stress, which may result in DNA damage. In addition, they have been found to be carcinogenic through their genotoxic properties. They have been shown to be present in various types of cancer, including cancer of the female reproductive system. Both metals have been recognized as metalloestrogens, which are important in hormone-related cancers. Participation in the oncogenesis of ovarian, endometrial and cervical cancer was analysed in detail, using the available research in this field. We emphasize their role as potential biomarkers in cancer risk and diagnosis as well as advancement of gynaecological malignancies.

## 1. Introduction

Various metals play a key role in the homeostasis of the human body. However, there is an important boundary between those necessary for living organisms in small amounts but revealing their toxic properties in higher concentrations, and those that do not play a special role in metabolism and are simply included in the mandatory toxic group. Cadmium (Cd) and lead (Pb) belong to the latter group. Since both Cd and Pb do not perform important biological functions, their deficiency does not have consequences, while their excess can cause various diseases [1]. Both belong to a larger group of elements—the so-called “heavy metals” [2]. This term, widely used in the scientific literature, although quite precise, is commonly defined as a group of elements with a density greater than 5 g/cm^3^ [3]. These elements are natural components of the Earth’s crust. As a result of a wide range of human activities, their natural geochemical cycles and biochemical balance have undergone colossal changes. Various industries, including transport and energy, as well as municipal waste management and soil fertilizers contribute to the anthropogenic causes of pollution and make Cd and Pb ubiquitous. Heavy metals are released into the air (including during combustion, mining and processing), into surface waters (through direct deposition, run-offs and releases from storage and transport) and into the soil (and thus into crops and other organisms through food). All of the above-mentioned processes and routes contribute to greater human exposure, occurring primarily through inhalation of polluted air or tobacco smoke and consumption of contaminated food, water, beverages and a wide range of other human goods, such as dietary supplements, drugs and cosmetics [4,5,6,7].

Researchers have emphasized that the concentrations of Pb and Cd are strongly and positively correlated with each other, regardless of the type of tissue analysed—endometrium, endocervix or polyps [8]. Previous studies have shown that the levels of these two metals in human body fluids (including seminal plasma) also show strong positive correlations [9]. Both studies support the idea that sources of exposure may be similar for both elements. Other common features of these elements is their ability to accumulate in flora and fauna (including humans) as well a long half-life [2]. Cumulative exposure (especially compared to short-term exposure) of these metals is particularly important in the aetiology of cancer [10].

Due to the global burden of cancer, there is an urgent need to identify risk factors. Particular attention was paid to trace elements and heavy metals as significant and, more promisingly, potentially modifiable environmental factors. Deficiency of trace elements that play an important role in maintaining homeostasis (e.g., cofactors) and the accumulation of some toxic metals may impair the host’s resistance to cancer. It is widely recognized that the multi-stage process of carcinogenesis involves both genetic and epigenetic changes, which are possible due to various environmental factors [1].

Metals are suspected to induce genotoxicity by multiple routes. The first important phenomenon associated with the initiation and progression of cancer is the interference with cellular redox regulation, which causes oxidative stress, i.e., a state in which the increased production of reactive oxygen species (ROS) is not sufficiently compensated by the antioxidant protection of the body, which is followed by DNA damage [11]. Both Cd and Pb are well known as oxidative stress inducers [12,13]. Despite the fact that they are redox-inactive metals, they exhibit toxic effects by binding to the sulfhydryl groups of proteins and depleting glutathione, thereby disrupting redox homeostasis [2].

In addition, they also inhibit DNA repair mechanisms. As a consequence, genome instability occurs, and critical mutations begin to accumulate, which further strengthens the first mechanisms. Metals cause changes in cell proliferation because they inhibit cell control by inactivating tumour suppressor genes or activating oncogenes in the cell cycle. Some studies support the induction of carcinogenesis through metal with the participation of epigenetic mechanisms [14]. DNA methylation and histone modification are essential epigenetic mechanisms to ensure proper control of gene expression. Tumour suppressor gene transcription is inhibited by hypermethylation in the promoter region, whereas oncogene activation has been linked to a decrease in DNA methylation. This explains the huge role of the epigenetic component in the process of carcinogenesis [15,16,17]. The significance of heavy metals in the development of cancer and the variation in their concentrations between normal and cancerous tissues are of great interest. The importance of heavy metals in the development of cancer and the variability of their concentrations in normal and neoplastic tissues is of great interest. However, the exact role of heavy metals in carcinogenesis and the mechanisms that contribute to the development of the disease are still not fully understood.

Due to cadmium and lead’s special properties, both can mimic the action of oestrogen. They are considered as endocrine disruptors; they play a particular role in hormone-dependant diseases, including malignancies of the reproductive system [18,19,20,21].

In this review, special attention was paid to the role of lead and cadmium in oncogenesis and the formation of gynaecological cancers. To gather all currently recognized information on the topic, we performed a search of the literature, including studies up to October 2022. We used PubMed, Cochrane Library, Web of Science and Embase databases. We assessed the information included in articles published in English. We used a combination of keywords related to lead, cadmium, heavy metals, cancer, and gynaecological malignancies. We searched for relevant articles and chose the applicable ones to accomplish the objective of our review, which is to show the existing and described associations between the Cd and Pb and ovarian, cervical and endometrial cancers.

## 2. Lead

Lead is a very durable metal, and due to its physicochemical properties, it has found application in various industrial conditions [22]. Its durability is determined by its long presence in the environment—in water, dust, soil, as well as in manufactured products containing lead [23]. Wastes from domestic, industrial and commercial sources can contain various metals and contaminate soil and water sources such as groundwater and rivers [24].

The WHO classified lead as a toxic element both to humans and animals [23,25]. Bones and tissues act as lead stores, and the toxic metal is transported to these by the blood due to its ability to bind with erythrocytes. Lead is constantly mobilized from bone tissue into the blood. About 40–70% of the Pb in the blood comes from the skeleton in adults exposed to Pb [26]. Blood lead concentrations reflect short-term exposure to lead. The half-life of Pb is 1–2 months; then, it is removed from soft tissues and blood. Because of the indication of long-term exposure, bone tissue has been postulated to be a better indicator of chronic lead toxicities because it contains the majority (95%) of adult lead deposits [27,28], which are removed much more slowly, with a half-life of years to decades. Urine excretion accounts for around 70% of Pb excretion, with faeces accounting for a smaller portion. Some is lost through sweat, hair and nails [29]. Lead can cross the placenta (and accumulate in the developing brain) and can be passed on to babies through breast milk [30]. In women, lead exposure is associated with several detrimental effects on women’s reproductive health. The following have been reported in clinical trials: spontaneous abortion, premature birth, foetal growth abnormalities, preterm delivery, low birth weight, pre-eclampsia, stillbirth, gestational diabetes and neonatal death [31]. Elevated blood levels are the most common way to identify lead toxicity. A blood level of 10 g/dL (0.48 mol/L) or higher is considered dangerous; it can affect many organs and can range from clear clinical signs to enigmatic biochemical ones [32,33]. This may result in neurological disorders, peripheral neuropathy and cognitive impairments [32]. Lead is a neurotoxic substance that interferes with the formation of synaptic connections in the cerebral cortex and is associated with behavioural and psychological problems in humans and other animals [34,35]. In adults, high lead intake is associated with potential development of nephropathy, hypertension and other disorders [32,33]. Occupational and environmental exposure to lead is not indifferent to the libido and impairs the synthesis and circulation of sex hormones and menstruation, additionally reducing fertility, delaying the time of conception and affecting the outcome of pregnancy [36].

### 2.1. Lead and Oxidative Stress

Under normal circumstances, free radicals and ROS are neutralized by the body’s antioxidant defences; however, when the body’s reserves secrete unopposed oxidants, it causes harmful effects and damage [2]. Oxidative stress is a key element in the aetiology of a wide spectrum of diseases, including chronic, vascular and immunological diseases, as well as atherosclerosis, neurodegeneration, aging processes, mutagenesis and carcinogenesis [37].

**Figure 1:**
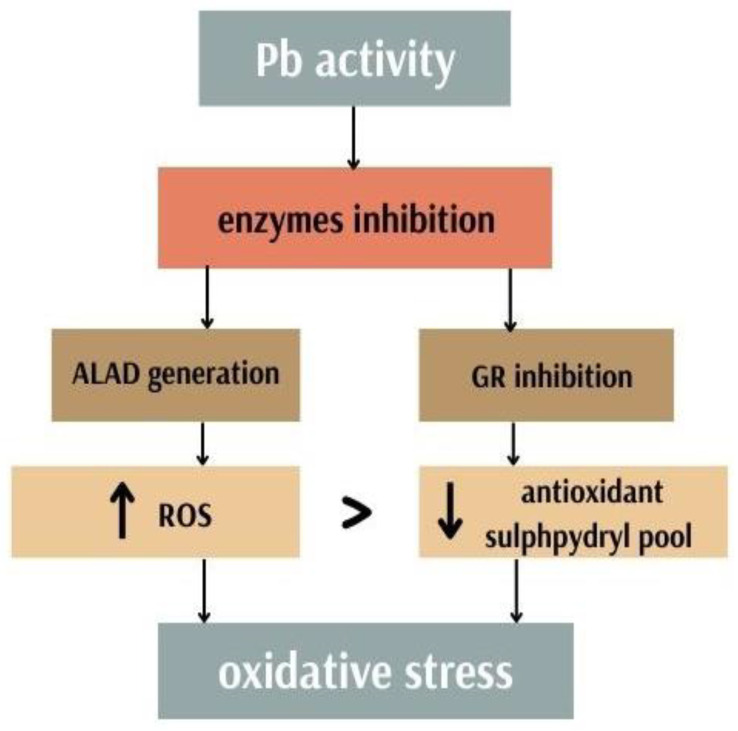
Lead activity and oxidative stress generation.

Lead causes excessive levels of oxidative stress, which damages cellular components in a similar way as other persistent hazardous metals (such as cadmium); see Figure 1. Lead is also known to cause lipid peroxidation. Nevertheless, this metal is a redox inactive. Due to this fact, Pb and do not possess the ability to easily undergo valance changes. The pathogenetic effects of lead are multifactorial. First, singlet oxygen, hydrogen peroxides and hydroperoxides as well as other ROS are produced. Second, the cellular pool of antioxidants is depleted [2]. Thus, two independent but connected mechanisms contribute to free radical damage caused by Pb [38]. These two mechanisms are interrelated, so when ROS levels increase on one side, antioxidant pools are depleted on the opposite side [39]. The δ-aminolevulinic acid substrate, known to induce ROS production, is increased by lead inhibition of delta-aminolevulinic acid dehydratase (ALAD). Lead-induced free radical damage is caused by these separate but connected pathways [2]. Pb directly interrupts the activity of enzymes, competitively inhibits the absorption of important trace elements and deactivates the sulfhydryl pools of antioxidants [40]. Lead mainly depletes the cellular antioxidant pool by attaching itself to the sulfhydryl groups of proteins and lowering glutathione levels [2]. Lead inhibits two specific enzymes—glutathione reductase (GR) and ALAD [41]. Lead has been shown to interfere with the cycle of converting oxidized glutathione (GSSG) to reduced glutathione (GSH), which lowers GSH levels. GR is the enzyme responsible for recycling GSH [42]. More than 90% of the body’s non-tissue sulphur pool is covered by glutathione, and lead primarily affects glutathione metabolism [43].

In addition to ROS, reactive nitrogen species (RNS) have also been shown to play a key role in lead toxicity, as demonstrated in a study of lead-induced hypertension in humans [44]. The relaxation factor derived from the endothelium is nitric oxide. Nitric oxide in vascular endothelial cells can be oxidized by ROS from lead exposure to form peroxynitrite (ONOO), a highly reactive ROS that can damage lipids and DNA. In animal studies, elevated blood pressure is caused by NO depletion after exposure to lead [45].

### 2.2. Lead and Carcinogenicity

Lead has been classified by IARC as a category 2B carcinogen, due to its proven carcinogenicity in animals [46]. The mechanism of lead-related carcinogenicity remains unclear, although there are studies that have shown that lead destabilizes the structure of DNA, inducing chromatin aggregation through histone-DNA cross-links [47]. Lead has been found to have a proliferative effect on liver cells in vivo as a mitogen [48]. This proliferation was found to be directly hyperplastic and associated with TNF-α [49]. A similar dose-dependent proliferative effect on renal tubular epithelial cells with occasional atypias was also observed in lead-exposed rats. Lower doses caused nephrotoxic effects [50,51].

Increased Pb concentration in toenails was also associated with the risk of pancreatic carcinogenesis [52]. In a prospective analysis of 4740 workers in Pb and Zn smelters, long-term and low exposure to these metals was shown to be a risk factor for lung cancer. A nine-fold increase in the probability of peritoneal and retroperitoneal malignancies has also been documented [53]. Additionally, patients with gallbladder cancer were found to have higher levels of lead in their tissues [54]. Moreover, exposure to Pb was linked to breast cancer because it acts as a metalloestrogen and activates the oestrogen receptor-α [18]. Concentrations of lead and cadmium in the scalp, hair and nails were also found to be higher in patients with lung and prostate cancer in comparison to those of healthy controls [55,56].

## 3. Cadmium

Cadmium is a heavy metal with an extremely long biological half-life [57]. Cadmium has no important biologic functions [58], but as a cumulative toxin is a metal of ongoing environmental and occupational concern, with a multitude of negative impacts [57]. Chronic low levels of exposure to cadmium have been associated with a higher risk of many diseases, including cancer, cardiovascular disease, bone damage, renal tubular disease, diabetes and obstructive pulmonary disease [59].

There are several routes of Cd uptake, which include the lungs, skin and digestive system [60]. Few sources of human exposure to cadmium make excellent use of these routes, posing a risk to human health. One of the most dangerous transition metals, Cd, is associated with air and water pollution and cigarette smoke [61]. It is worth noting that cadmium makes up 0.1 parts per million (ppm) of the Earth’s crust [62]; historically, exposure has been inversely correlated with environmental occurrence. However, over the last century, the use of cadmium in manufacturing has increased dramatically, significantly changing the risk of exposure to this rare element [63]. Numerous studies have been conducted on the harmful effects of Cd, with a major focus on occupational exposure [64], including employment in the primary metals industry [57].

Still, smoking is the main source of Cd intake. Tobacco plants naturally accumulate relatively high concentrations of cadmium in their leaves. Each cigarette contains 1 to 2 μg of Cd. Smokers can consume up to twice as much cadmium per day than non-smokers when they smoke 20 cigarettes per day, resulting in an annual cadmium accumulation of 0.5 mg [65].

Although gastrointestinal absorption from food is typically much less effective than from air or water due to the fact that Cd binds to food components, food is still the main source of Cd for occupationally unexposed people [66]. Estimated global dietary intakes of cadmium range from 10 to 40 micrograms per day in pollution-free areas, and up to several hundred micrograms in cadmium-contaminated areas [67].

Compared to less than 10% of Cd absorbed with food, it is estimated that 10 to 40% of Cd can be absorbed by inhalation [68]. Food also remains the major source of Cd for non-smokers [67].

### 3.1. Cadmium and Zinc

Zinc, being one of the most crucial trace elements for humans, acts as a catalyst regulatory and structural ion. As a trace metal, it plays a key role in maintaining homeostasis and the proper functioning of the immune system. It also protects the human body against the adverse effects of oxidative stress and is necessary in apoptosis and the process of aging [69]. More than 300 enzymes use it as a cofactor, although its primary function is to stabilize the structure of proteins, especially signalling enzymes [70,71]. Zinc is believed to potentially inhibit tumour growth due to its antioxidant properties [37]. The negative effects of cadmium often result from interference with various zinc-mediated metabolic processes. The usage of zinc in treatment can reduce or abolish the toxic effects of cadmium, which supports their specific relationship [66]. Being structurally similar to zinc, Cd competes with Zn for protein binding sites and can replace them. It has been hypothesized that the toxicity of cadmium results from its role as a key Zn cofactor in many critical enzyme systems [72].

In one of the studies on the effect of exposure to cadmium in rat heart tissue, reduced activity of superoxide dismutase (SOD) was demonstrated [73]. Cadmium poisoning changes the activity of antioxidant enzymes, one of which is Zn-SOD, which was also demonstrated in another study on rats [74]. Zinc is one of the components of SOD—an enzyme that catalyses the dismutation of superoxide radicals to hydrogen peroxide. Reducing its activity may cause oxidative stress and lead to carcinogenesis and tumour progression [75,76].

It has been reported that one of the key mechanisms by which cadmium antagonizes zinc may be the result of its replacement in the DNA-binding domain of the Zn finger, and this may be how Cd causes not only toxicity but also cancer [77]. Zinc finger proteins (Zfp) are the largest family of transcription factors in the genome of humans. They are sequence-specific DNA-binding proteins [78]. Zinc is the main structural component of Zfp and is necessary to ensure their stability [79] They are present in almost half of human transcription factors, making them major determinants of network and regulatory processes [80].

Moreover, the inhibition of repair is attributed to displacement of Cd(II) ions due to the fact that Mg and Zn, which are cofactors of DNA polymerase, effectively protect against carcinogenesis in vivo [81]. In addition to the accumulation of potentially dangerous and unnecessary trace metals, an imbalance in the trace metal composition, known to be essential for homeostasis, can cause disease. This may suggest that the host’s resistance to carcinogenic stress may be impaired by the lack of vital trace metals, which act as enzyme cofactors [82].

### 3.2. Cadmium and Metalothioneins

Cadmium accumulates in tissues because there is no mechanism for excretion of Cd for humans. Cadmium in the renal cortex has a half-life of 20–35 years. Cadmium accumulates mainly in the liver and kidneys. The next largest metal deposits are the pancreas and lungs. In the body, Cd is mainly associated with metallothioneins (MT) [60]. Proteins called MTs, which bind to low-molecular-weight metals, are expected to sequester the metal with high affinity [83]. Due to their nature of intracellular polypeptides, MTs have an extraordinary ability to bind to metal ions, including essential metals, as well as harmful and heavy metals such as cadmium and lead [82]. Immunohistochemically detectable overexpression of MT has been shown in various cancers, particularly breast cancer. They seem to play a homeostatic role in the control and detoxification of these metals [84].

### 3.3. Cadmium and Antioxidants

**Figure 2:**
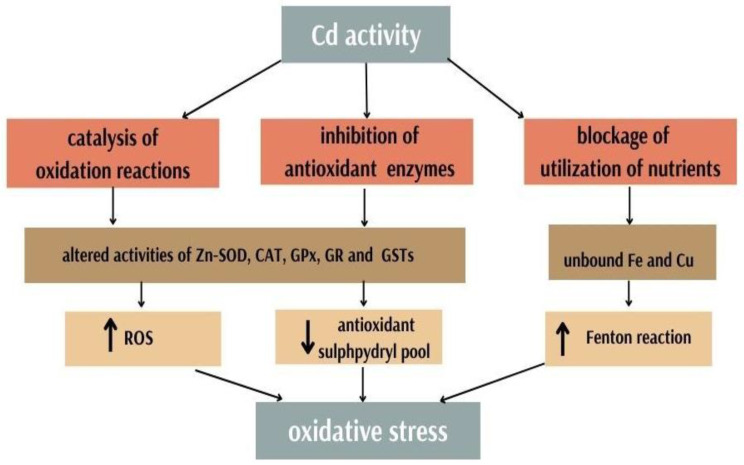
Cadmium activity and oxidative stress generation.

Disruption of metal ion homeostasis can lead to oxidative stress [2]. Cd is an important factor that strongly contributes to the disruption of redox homeostasis in the cell. Its toxic effect is due to several mechanisms: generation of tissue damage by free radicals, as it can be a catalyst for oxidation reactions, inhibition of enzymes of the antioxidant system, and blocking the use of nutrients (Figure 2) [58].

Cadmium itself is not able to directly generate free radicals, but its action results in increased indirect production of ROS and RNS with the participation of superoxide radicals, hydroxyl radicals and nitric oxide [85]. The generation of hydrogen peroxide from non-radical particles has been confirmed, which in turn can be a relevant source of radicals through Fenton reaction [86]. Cadmium poisoning leads to a substantial increase in the concentration of lipid peroxides and changes the activity of antioxidant enzymes such as Zn-SOD, catalase (CAT), glutathione peroxidase (GPx), glutathione reductase (GR) and glutathione S-transferase (GST) stocks in rats [74]. Cd also depletes sulfhydryl groups associated with proteins [77]. One of the studies on the consequences of exposure to cadmium (exposure in drinking water in rat heart tissue) showed a considerable increase in the markers of oxidative stress—malondialdehyde (MDA) and lipoperoxides—and a decrease in SOD and GPx activity [73].

In addition, studies also reveal that under the conditions of exposure to cadmium and the excessive amount of ROS caused by its presence, the number of cells with single-strand DNA breaks and cellular DNA damage were significantly elevated in the exposed groups compared to the control groups [87]. Another mechanism of the indirect role of Cd in the formation of free radicals consisted of the replacement of iron and copper in various cytoplasmic and membrane proteins (such as apoferritin and ferritin). This increases the amount of unbound free or weakly chelated Cu and Fe ions involved in oxidative stress through Fenton chemistry. Thanks to this reaction, the copper displaced from the binding site can catalyse the decomposition of hydrogen peroxide. The replacement of Fe and Cu by cadmium may be a good explanation for the increased toxicity induced by Cd [88].

The toxic effects of cadmium are known to occur intracellularly, mainly as a result of damage caused by free radicals. These effects are particularly damaging to the lungs, heart, bones, kidneys, central nervous system and reproductive system. However, the exact mechanisms are not yet fully understood [89].

### 3.4. Cadmium and Cancerogecity

As stated by the International Agency for Research on Cancer (IARC), there is sufficient evidence of human carcinogenicity of cadmium and cadmium compounds, which have been identified as Group 1 human carcinogens [66]. Cd has already been identified in several malignancies [24]. In plant, animal and human cells, Cd causes DNA strand breakage, sister chromatid exchange, chromosomal aberrations and oxidative damage [90,91]. Moreover, Cd enhances the mutagenic properties of UV light [81]. The induction of oxidative DNA damage and interaction with DNA repair processes are responsible for the genotoxic properties of Cd. Two mechanisms responsible for DNA repair, nucleotide excision repair (NER) (the master repair system) and repair of oxidative modifications of DNA bases, are disrupted by Cd(II) ions [81].

An interesting explanation of cadmium-induced carcinogenicity in relation to cell adhesion suggests that E-cadherin, being a transmembrane glycoprotein, plays an important role in cell–cell adhesion and has the ability to attach Cd to Ca(II) binding regions, resulting in a conformation change of the glycoprotein. Disturbances in cell–cell adhesion caused by this heavy metal seem to be a possible explanation for the induction and promotion of cancer in some cases [92].

Cd is a strong carcinogen that may cause prostate, lung and gastrointestinal (particularly pancreatic and kidney) cancer. Cigarette smoke acts synergistically in the process of carcinogenesis and intensifies its effects [93,94]. The incidence of cancer (especially of the lungs) has been extensively studied in the polluted environment in the vicinity of zinc smelters. The study outcome revealed a relationship between cancer risk and environmental exposure to Cd, as evidenced by 24 h urinary excretion, a result that remained consistent after adjusting for gender, age and smoking [95]. In addition to lung cancer, associations between urinary cadmium levels and leukaemia mortality in both sexes have also been suggested [96].

## 4. Lead, Cadmium and Gynaecological Malignancies

17β—estradiol (E2) and other steroidal oestrogens have key physiological effects for many tissues; exposure to oestrogens is a known risk factor, which is especially important in gynaecological cancers, as they are often hormonal in origin [97,98,99]. Chemical substances inducing estrogenic activity can be divided into several groups, such as oestrogens (e.g., 17β-estradiol), xenoestrogens (e.g., Bisphenol-A), phytoestrogens (e.g., genistein) and metalloestrogens (cadmium and lead) [100]. Inorganic heavy metal ions that attach to oestrogen receptors and induce their subsequent activation are called “metalloestrogens”. Cd and Pb are among these; they have estrogenic properties and have been found to produce a pronounced oestrogen-like effect [19,101]. Metalloestrogens can be divided into two distinct subclasses: oxanions and divalent cations Cd and Pb. Metalloestrogens and metalloids (semi-metals) possess the ability to activate the oestrogen receptor (ER) [98]. Two ER isoforms—Erα and Erβ—can mediate many of the effects of oestrogens. Erα activation results in the mitogenic effects associated with this hormone, whereas Erβ activation results in the opposite, anti-mitogenic effects [102]. It has been hypothesized that environmental pollutants resembling the action of oestrogens cause reproductive system disorders [97].

Because Pb acts as a metalloestrogen by activating the oestrogen receptor-α, it has been linked to breast cancer [18]. It has been found that the toxic metal induces cell proliferation and increases the transcription and expression of genes regulated by oestrogens, causing oestrogen-dependent breast cancer [103]. Cd has also been found to have oestrogen-like capacity and functionally acts as an endocrine disruptor by mimicking the effects of oestrogen [19,20,21]. In this study on ER levels and oestrogen-induced responses in Cd-induced human breast cancer cells, the metal was shown to significantly decrease ER levels, decrease ER mRNA and increase progesterone receptors, which are typical estrogenic effects [19]. Another study aimed to gain insight into the mechanism by which Cd activates ER-α. Researchers were able to provide evidence that the oestrogen receptor activation occurs through the interaction of a high affinity metal with the receptor’s hormone-binding domain. The analysis of the mutations helped in identification of a few amino acids as potential metal interaction sites, implying that ER-α activation by Cd occurs by forming a coordination complex in the hormone-binding domain of the receptor [20]. Environmentally significant doses of cadmium induced a few very typical oestrogen responses in rats. Increase in uterine weight and progesterone receptor expression, endometrial hypertrophy and hyperplasia, as well as mammary gland growth and development, were observed [104].

There are not many studies addressing the correlation of Cd and Pb with gynaecological malignancies, but some of the results from those commonly available are summarized below.

### 4.1. Cervical Cancer

Cervical cancer was the most common and deadly gynaecological malignancy in the world in 2020 [105]; it is disproportionately common (>80%) in the underdeveloped world [106]. Early sexual initiation, specific sexual behaviours such as having multiple partners, having sex at a young age, infrequent condom use, multiple pregnancies, chlamydial infections and HIV-related immunosuppression, which is associated with a higher risk of HPV (human papilloma virus) infection, are blamed for the growing trend of the disease in developing countries [107]. Smoking, multiparity and long-term use of oral contraceptives can double or triple the risk of precancerous disease and cancer in women infected with carcinogenic HPV types [108,109,110]. Persistent infection with one of the approximately 15 genotypes of carcinogenic HPV causes almost all cases of cervical cancer—both squamous cell carcinoma and cervical adenocarcinoma [111].

In one study, the Cd content of scalp hair samples was significantly higher in the hair of cancer patients compared to women in the reference group. The result was obtained in cervical and ovarian cancer [112]. Another study was conducted on patients with various stages of cervical cancer. Parameters were compared between blood samples taken before and after radiotherapy. In this study, plasma Cd increased in the cancer stage 3 and 4 groups, while the stage 1 and 2 patient groups showed no marked change. Cd levels fell to near normal levels after 30 days of radiotherapy. The authors speculated that the observed increase in the level of Cd in patients with stage 3 and 4 may result from the specific effect of advanced tumor mass growth. In their opinion, another reasonable speculation is that the accumulation of Cd may be the reason for the reduction of zinc levels in advanced stages of cervical cancer due to the structural similarity between them [113].

Studies on Pb showed higher Pb levels associated with higher risk of cervical cancer compared to HPV-negative/non-cancerous individuals (adjusted for age effect). The study characterized the potential cancerous role of lead (Pb) as a common environmental toxicant for CIN (cervical intraepithelial neoplasia) outcomes. The finding suggests a direct significant relationship between Pb accumulation and the existence of CIN. Lead concentration was quantified using an atomic absorption spectrometer in liquid cytological samples. However, the authors themselves stated that their results and conclusions from inductive thinking should be treated with caution, because their cases concerned only potentially premalignant CIN transformation; it is worth noting that their study included only three cases of CIN [114]. However, another similar study with a larger sample followed, providing better statistical power. Researchers assessed and evaluated the relationship between Pb concentration in women suffering from CIN associated with HPV genotypes compared to non-HPV/non-cancerous outcomes. These studies provide unequivocal evidence of elevated Pb levels accumulated in cervical neoplastic tissue. They also focused on finding any relationship between HPV and Pb genotypes, but there was no significant difference [8].

### 4.2. Endometrial Cancer

Endometrial cancer is the second most common among gynaecological malignancies [115]. Most cases are detected after menopause [116]. Typical risk is more common in developed countries; however, though endometrial cancer is less common in less developed countries, the mortality rate is much higher. The most common—type 1 lesions—are usually hormone-sensitive, low-stage, and have an excellent prognosis, while type 2 tumours are mostly high-grade and recurrent tumours, even at an early stage. The endometrium undergoes constant structural modification in response to the cyclical changes in oestrogen and progesterone levels during the menstrual cycle. Long-term, non-contradictory exposure to oestrogen leads to endometrial hyperplasia, which increases the risk of developing atypical hyperplasia and ultimately type 1 endometrial cancer [117]. The involvement of most risk factors in its development can be explained by the phenomenon of unopposed-oestrogen action. This includes being overweight, usage of tamoxifen, nulliparity and hormone replacement therapy with insufficient time for progesterone compound addition as important risk factors [118,119,120,121,122]. Great multiparity and pregnancy, birth control pills containing oestrogens with progestogens, and smoking reduce the risk of endometrial cancer [123,124,125,126]. Due to the large number of blood vessels of the endometrium and the endocervix, they may be sensitive to many substances supplied by blood, both endogenous (such as hormones) and exogenous (such as toxic heavy metals, which may accumulate with prolonged exposure) [127].

Elevated levels of Cd have been observed in neoplastic endometrial tissues [8]. Urine Cd studies have also provided some evidence of an association between increased uCd levels and higher endometrial cancer [96]. Cd from food has also been identified as a potential risk factor for endometrial cancers in postmenopausal women, but there is no consensus among researchers. Some studies do not indicate that our estimated dietary intake of Cd is associated with hormone-related cancers in women [128]. However, authors offer several possible explanations for why their results were inconclusive, for example, misclassification in the assessment of dietary Cd may have weakened the observed associations [129]. However, other dietary studies have linked elevated levels of Cd in food to the risk of endometrial cancer. Statistical significance of long-term cadmium intake and a higher risk of cancer of the endometrium was proven in all women. These outcomes support the hypothesis that Cd induces estrogenic effects, which may result in a greater risk of hormone-related malignancies [130]. A population-based case-control study of women in the Midwestern United States found a statistically significant positive association between urinary Cd levels and endometrial cancer risk. A doubling of cadmium exposure increased the risk of endometrial cancer by 22%. These findings show that Cd may increase the risk of endometrial cancer, perhaps due to the effects of oestrogen [131]. The goal of another researcher was to conduct a study that would answer the question of how changes in blood Cd levels behave during six cycles of chemotherapy in patients with genital cancer, taking in account the treatment stage. In endometrial cancer, concentrations increased slightly in the first cycle of chemotherapy but decreased in the third and remained at a similar level in the sixth. In-depth study of the collected data showed the interaction between the stage of chemotherapy and the type of cancer affected the concentration of Cd. This was considerably affected by the cancer type as well as the chemotherapy cycle used. Insightful analysis can prove useful in better understanding the physiologic processes resulting from chemotherapy. Researchers point out that further studies on trace element monitoring may determine the efficacy of cancer treatment, thereby improving treatment outcomes and extending patient survival time [132]. Compared to histologically normal tissues, endometrial cancer, hyperplasia and CIN demonstrated relevantly increased toxic metal concentration—not only for cadmium but also lead. Understanding of the existence, function and diseases of metals in the female reproductive system is facilitated by the findings of this study [8]. As noted in another study, hyperplasia of endometrial tissue, considered one of the risk factors for the development of cancer [133], also showed substantially increased concentrations of lead compared to healthy tissue. Elevated Pb concentration in these tissues may contribute to the advancement of lesions and tumorigenesis, given that it may increase cellular levels of reactive oxygen species and cause genetic imbalance [13].

### 4.3. Ovarian Cancer

Among gynaecological malignancies, ovarian cancer ranks second in terms of mortality [134], mainly due to relapse and chemoresistance [135]. There are several genes that have been found to be connected to hereditary ovarian cancer. About 23% of ovarian cancer cases are thought to have a hereditary predisposition. Germline mutations in the BRCA1 or BRCA2 genes, which account for 20–25% of high-grade serous ovarian cancer, are the most commonly inherited disorder [136]. Other important factors are menstrual and hormonal factors (such as early menstrual age and late menopause), use of hormone replacement therapy during menopause, and high BMI. Unambiguously higher parity and the usage of oral contraceptives lower the risk of ovarian cancer [137]. The abovementioned study of Cd in hair samples of female cancerous patients and their healthy female counterparts clearly advocates for a strong association of ovarian and cervical cancers with elevated Cd concentration [112]. Research confirms the relationship between higher urinary Cd concentrations and an increase in the incidence of ovarian cancer [96]. Some studies suggest that exposure to dietary Cd is unlikely to have a significant effect on the development of ovarian cancer [128,138]. An interesting discovery was made in the study of changes in the concentration of Cd in the blood during six cycles of chemotherapy. In ovarian cancer, Cd levels increased in the third cycle of chemotherapy and then decreased slightly in the sixth cycle. The results of the study indicate that the level of Cd was significantly dependent on the cancer type and the course of chemotherapy [132]. Pb and its concentrations in ovarian tissue were found to be increased in both malignant and borderline tissues compared to healthy ovaries. Lead concentrations in malignant tissues, borderline papillary projections, and capsular tissue samples were not different. This study concluded that Pb accumulation in the tissues of the ovary was related, with borderline and malignant proliferation of the superficial epithelium [10].

## 5. Conclusions

Both lead and cadmium play an important role in the oncogenesis of gynaecological cancers due to their ability to generate oxidative stress, genotoxicity and metalloestrogenic properties. Undoubtedly, further research into the pathogenesis mechanisms of various malignancies is needed, as there are still many gaps in our understanding of carcinogenesis and the involvement of heavy metals in this process. Gynaecological cancers are one of the most common causes of death among women; thus, it is important to focus on this topic. While the environmental pollutants mimicking the effects of oestrogen have been suggested to contribute to the high rate of hormone-related cancers, little additional data exist. In particular, the scientific literature on Pb in genital cancer is limited. With this in mind, there is a strong need for trials with larger patient populations and multicentre trials to create studies with greater statistical power. Cd and Pb appear to be modifiable risk factors for cancer, making a compelling case for intensified efforts to eliminate their exposure. A deeper understanding of these heavy metals may prove to be a great diagnostic clue, and they may have a potential role as biomarkers for cancer risk or advancement.

## References

[1] World Health Organization, Food and Agriculture Organization of the United Nations, International Atomic Energy Agency (1996). Trace Elements in Human Nutrition and Health.

[2] Jomova K., Valko M. (2011). Advances in metal-induced oxidative stress and human disease. Toxicology.

[3] Duffus J.H. (2002). ‘Heavy Metals’—A Meaningless Term? (IUPAC Technical Report). Pure Appl. Chem..

[4] Szyczewski P., Siepak J., Niedzielski P., Sobczyński T. (2009). Research on Heavy Metals in Poland. Pol. J. Environ. Stud..

[5] Callan A., Hinwood A., Devine A. (2014). Metals in commonly eaten groceries in Western Australia: A market basket survey and dietary assessment. Food Addit. Contam. Part A.

[6] Iwegbue C.M.A., Ojelum A.L., Bassey F.I. (2014). A survey of metal profiles in some traditional alcoholic beverages in Nigeria. Food Sci. Nutr..

[7] Rzymski P., Niedzielski P., Kaczmarek N., Jurczak T., Klimaszyk P. (2015). The multidisciplinary approach to safety and toxicity assessment of microalgae-based food supplements following clinical cases of poisoning. Harmful Algae.

[8] Rzymski P., Niedzielski P., Rzymski P., Tomczyk K., Kozak L., Poniedzia1ek B. (2016). Metal accumulation in the human uterus varies by pathology and smoking status. Fertil. Steril..

[9] Mínguez-Alarcón L., Mendiola J., Roca M., López-Espín J.J., Guillén J.J., Moreno J.M., Moreno-Grau S., Martínez-García M.J., Vergara-Juárez N., Elvira-Rendueles B. (2012). Correlations between Different Heavy Metals in Diverse Body Fluids: Studies of Human Semen Quality. Adv. Urol..

[10] Canaz E., Kilinc M., Sayar H., Kiran G., Ozyurek E. (2017). Lead, selenium and nickel concentrations in epithelial ovarian cancer, borderline ovarian tumor and healthy ovarian tissues. J. Trace Elem. Med. Biol..

[11] Ames B.N., Shigenaga M.K. (1992). Oxidants are a major contributor to aging. Ann. N. Y. Acad. Sci..

[12] Reuter S., Gupta S.C., Chaturvedi M.M., Aggarwal B.B. (2010). Oxidative stress, inflammation, and cancer: How are they linked?. Free Radic. Biol. Med..

[13] Matović V., Buha A., Ðukić-Ćosić D., Bulat Z. (2015). Insight into the oxidative stress induced by lead and/or cadmium in blood, liver and kidneys. Food Chem. Toxicol..

[14] Beyersmann D., Hartwig A. (2008). Carcinogenic metal compounds: Recent insight into molecular and cellular mechanisms. Arch. Toxicol..

[15] Bello M., Alonso M., Amiñoso C., Anselmo N.P., Arjona D., Gonzalez-Gomez P., Lopez-Marin I., de Campos J.M., Gutierrez M., Isla A. (2004). Hypermethylation of the DNA repair gene MGMT: Association with TP53 G:C to A:T transitions in a series of 469 nervous system tumors. Mutat. Res. Mol. Mech. Mutagen..

[16] Park I.Y., Sohn B.H., Yu E., Suh D.J., Chung Y.H., Lee J.H., Surzycki S.J., Lee Y.I. (2007). Aberrant Epigenetic Modifications in Hepatocarcinogenesis Induced by Hepatitis B Virus X Protein. Gastroenterology.

[17] Patel A.C., Anna C.H., Foley J.F., Stockton P.S., Tyson F.L., Barrett J., Devereux T.R. (2000). Hypermethylation of the p16 (Ink4a) promoter in B6C3F1 mouse primary lung adenocarcinomas and mouse lung cell lines. Carcinog..

[18] González-González A., Mediavilla M.D., Sánchez-Barceló E.J. (2018). Melatonin: A Molecule for Reducing Breast Cancer Risk. Molecules.

[19] Garcia-Morales P., Saceda M., Kenney N., Kim N., Salomon D., Gottardis M., Solomon H., Sholler P., Jordan V., Martin M. (1994). Effect of Cadmium on Estrogen Receptor Levels and Estrogen-induced Responses in Human Breast Cancer Cells. J. Biol. Chem..

[20] Stoica A., Katzenellenbogen B.S., Martin M.B. (2000). Activation of Estrogen Receptor-by the Heavy Metal Cadmium. Mol. Endocrinol..

[21] Brama M., Gnessi L., Basciani S., Cerulli N., Politi L., Spera G., Mariani S., Cherubini S., D’Abusco A.S., Scandurra R. (2007). Cadmium induces mitogenic signaling in breast cancer cell by an ER-dependent mechanism. Mol. Cell. Endocrinol..

[22] Brännvall M.-L., Bindler A.R., Renberg I., Emteryd O., Bartnicki J., Billström K. (1999). The Medieval Metal Industry Was the Cradle of Modern Large-Scale Atmospheric Lead Pollution in Northern Europe. Environ. Sci. Technol..

[23] Kumar A., Clark C.S. (2009). Lead loadings in household dust in Delhi, India. Indoor Air.

[24] Hayes R. (1997). The carcinogenicity of metals in humans. Cancer Causes Control.

[25] (1995). Environmental Health Criteria 165 INORGANIC LEAD.

[26] Smith D.R., Osterloh J.D., Flegal A.R. (1996). Use of Endogenous, Stable Lead Isotopes to Determine Release of Lead from the Skeleton. Environ. Health Perspect..

[27] Landrigan P.J., Todd A.C. (1994). Direct Measurement of Lead in Bone A Promising Biomarker. JAMA.

[28] Somervaille L.J., Chettle D.R., Scott M.C., Tennant D.R., McKiernan M.J., Skilbeck A., Trethowan W.N. (1988). In vivo tibia lead measurements as an index of cumulative exposure in occupationally exposed subjects. Br. J. Ind. Med..

[29] Leggett R.W. (1993). An age-specific kinetic model of lead metabolism in humans. Environ. Health Perspect..

[30] Ettinger A.S., Téllez-Rojo M.M., Amarasiriwardena C., Bellinger D., Peterson K., Schwartz J., Hu H., Hernández-Avila M. (2004). Effffect of Breast Milk Lead on Infant Blood Lead Levels at 1 Month of Age. Environ. Health Perspect..

[31] León O.L.L., Pacheco J.M.S. (2020). Effects of Lead on Reproductive Health. Lead Chemistry.

[32] Patrick L. (2006). Lead toxicity, a review of the literature. Part 1: Exposure, evaluation, and treatment. Altern. Med. Rev..

[33] Tyroler H.A. (1988). Epidemiology of hypertension as a public health problem: An overview as background for evaluation of blood lead-blood pressure relationship. Environ. Health Perspect..

[34] Goldstein G.W. (1992). Neurologic concepts of lead poisoning in children. Pediatr. Ann..

[35] Needleman H.L., Rabinowitz M., Leviton A., Linn S., Schoenbaum S. (1984). The Relationship Between Prenatal Exposure to Lead and Congenital Anomalies. JAMA.

[36] Kumar S. (2018). Occupational and Environmental Exposure to Lead and Reproductive Health Impairment: An Overview. Indian J. Occup. Environ. Med..

[37] Das K.C., White C.W. (2002). Redox systems of the cell: Possible links and implications. Proc. Natl. Acad. Sci. USA.

[38] Ercal N., Gurer-Orhan H., Aykin-Burns N. (2001). Toxic metals and oxidative stress part I: Mechanisms involved in metal-induced oxidative damage. Curr. Top. Med. Chem..

[39] Gurer H., Ercal N. (2000). Can antioxidants be beneficial in the treatment of lead poisoning?. Free Radic. Biol. Med..

[40] Patrick L. (2006). Lead toxicity part II: The role of free radical damage and the use of antioxidants in the pathology and treatment of lead toxicity. Altern. Med. Rev. J. Clin. Ther..

[41] Beyer W.N., Audet D.J., Heinz G.H., Hoffman D.J., Day D. (2000). Relation of Waterfowl Poisoning to Sediment Lead Concentrations in the Coeur d’Alene River Basin.

[42] Sugawara E., Nakamura K., Miyake T., Fukumura A., Seki Y. (1991). Lipid peroxidation and concentration of glutathione in erythrocytes from workers exposed to lead. Br. J. Ind. Med..

[43] Hunaiti A., Soud M. (2000). Effect of lead concentration on the level of glutathione, glutathione S-transferase, reductase and peroxidase in human blood. Sci. Total Environ..

[44] Valko M., Leibfritz D., Moncol J., Cronin M.T.D., Mazur M., Telser J. (2007). Free radicals and antioxidants in normal physiological functions and human disease. Int. J. Biochem. Cell Biol..

[45] Vaziri N.D., Wang X.Q., Oveisi F., Rad B. (2000). Induction of oxidative stress by glutathione depletion causes severe hypertension in normal rats. Hypertension.

[46] Vainio H., Hemminki K., Wilbourn J. (1985). Data on the carcinogenicity of chemicals in the IARC monographs programme. Carcinogenesis.

[47] Rabbani-Chadegani A., Abdosamadi S., Fani N., Mohammadian S. (2009). A comparison of the effect of lead nitrate on rat liver chromatin, DNA and histone proteins in solution. Arch. Toxicol..

[48] Kanduc D., Rossiello M.R., Aresta A., Quagliariello E., Cavazza C., Farber E. (1991). Transitory DNA hypomethylation during liver cell proliferation induced by a single dose of lead nitrate. Arch. Biochem. Biophys..

[49] Shinozuka H., Ohmura T., Katyal S.L., Zedda A.I., Ledda-Columbano G.M., Columbano A. (1996). Possible roles of nonparenchymal cells in hepatocyte proliferation induced by lead nitrate and by tumor necrosis factor α. Hepatology.

[50] Choie D.D., Richter G.W. (1972). Cell Proliferation in Rat Kidneys After Prolonged Treatment with Lead. Am. J. Pathol..

[51] Herbertson B.M., King A.J., Allen J. (1987). Epithelial cell proliferation in the rat urinary system induced by parenteral injection of lead salts. Br. J. Exp. Pathol..

[52] Amaral A.F.S., Porta M., Silverman D.T., Milne R.L., Kogevinas M., Rothman N., Cantor K.P., Jackson B.P., Pumarega J.A., López-Jiménez T. (2011). Pancreatic cancer risk and levels of trace elements. Gut.

[53] Cocco P.L., Carta P., Belli S., Picchiri G.F., Flore M.V. (1994). Mortality of Sardinian lead and zinc miners: 1960-88. Occup. Environ. Med..

[54] Basu S., Singh M.K., Singh T.B., Bhartiya S.K., Singh S.P., Shukla V.K. (2013). Heavy and trace metals in carcinoma of the gallbladder. World J. Surg..

[55] Qayyum M.A., Shah M.H. (2014). Comparative Study of Trace Elements in Blood, Scalp Hair and Nails of Prostate Cancer Patients in Relation to Healthy Donors. Biol. Trace Element Res..

[56] Qayyum M.A., Shah M.H. (2014). Comparative assessment of selected metals in the scalp hair and nails of lung cancer patients and controls. Biol. Trace Element Res..

[57] Sarkar B. (2002). Heavy Metals in the Environment.

[58] Yaman M., Kaya G., Simsek M. (2007). Comparison of trace element concentrations in cancerous and noncancerous human endometrial and ovary tissues. Int. J. Gynecol. Cancer.

[59] Larsson S.C., Wolk A. (2015). Urinary cadmium and mortality from all causes, cancer and cardiovascular disease in the general population: Systematic review and meta-analysis of cohort studies. Leuk. Res..

[60] Hamer D.H. (1986). Metallothionein. Annu. Rev. Biochem..

[61] Satarug S., Baker J.R., Urbenjapol S., Haswell-Elkins M., Reilly P.E., Williams D.J., Moore M.R. (2002). A global perspective on cadmium pollution and toxicity in non-occupationally exposed population. Toxicol. Lett..

[62] Wedepohl K.H. (1995). The composition of the continental crust. Geochim. Cosmochim. Acta.

[63] Cadmium Statistics and Information | U.S. Geological Survey. https://www.usgs.gov/centers/national-minerals-information-center/cadmium-statistics-and-information.

[64] Chen C.-L., Hsu L.-I., Chiou H.-Y., Hsueh Y.-M., Chen S.-Y., Wu M.-M., Chen C.-J., Blackfoot Disease Study Group (2004). Ingested arsenic, cigarette smoking, and lung cancer risk: A follow-up study in arseniasis-endemic areas in Taiwan. JAMA.

[65] ATSDR (2012). Toxicological Profile for Cadmium.

[66] IARC Working Group on the Evaluation of Carcinogenic Risks to Humans (1993). Beryllium, Cadmium, Mercury, and Exposures in the Glass Manufacturing Industry.

[67] Cuypers A., Plusquin M., Remans T., Jozefczak M., Keunen E., Gielen H., Opdenakker K., Nair A.R., Munters E., Artois T. (2010). Cadmium stress: An oxidative challenge. Biometals.

[68] (1992). Environmental Health Criteria 134 Cadmium.

[69] Hasapis C.T., Loutsidou A.C., Spiliopoulou C.A., Stefanidou M.E. (2012). Zinc and human health: An update. Arch. Toxicol..

[70] Michalczyk K., Cymbaluk-Płoska A. (2020). The Role of Zinc and Copper in Gynecological Malignancies. Nutrients.

[71] Beyersmann D. (2002). Homeostasis and Cellular Functions of Zinc. Mater. Werkst..

[72] Tietz N.W., Hirsch E.F., Neyman B. (1957). Spectrographic study of trace elements in cancerous and noncancerous human tissues. J. Am. Med. Assoc..

[73] Novelli E.L.B., Marques S.F.G., Almeida J.A., Diniz Y.S., Faine L.A., Ribas B.O. (2000). Toxic mechanism of cadmium exposure on cardiac tissue. Toxic Subst. Mech..

[74] Ognjanovic B., Pavlovic S., Maletić S.D., Zikić R.V., Stajn A.S., Radojicić R.M., Saicic Z., Petrović V.M. (2003). Protective Influence of Vitamin E on Antioxidant Defense System in the Blood of Rats Treated with Cadmium. Physiol. Res..

[75] Wang T., Liu B., Qin L., Wilson B., Hong J.-S. (2004). Protective effect of the SOD/catalase mimetic MnTMPyP on inflammation-mediated dopaminergic neurodegeneration in mesencephalic neuronal-glial cultures. J. Neuroimmunol..

[76] Liaw K.-Y., Lee P.-H., Wu F.-C., Tsai J.-S., Lin-Shiau S.-Y. Zinc, Copper, and Superoxide Dismutase in Hepatocellular Carcinoma. https://search.ebscohost.com/login.aspx?direct=true&profile=ehost&scope=site&authtype=crawler&jrnl=00029270&AN=16443985&h=WDjgonjb%2Fw7pLx0LnGUYkMRt9kGl2FBHk9Qb3qe9NOz4EgqVnBYszPnXvBYssnaVz7LM5IV05vw%2FOIofey6KrQ%3D%3D&crl=c.

[77] Stohs S.J., Bagchi D., Hassoun E., Bagchi M. (2001). Oxidative mechanisms in the toxicity of chromium and cadmium ions. J. Environ. Pathol. Toxicol. Oncol..

[78] Lander E.S., Linton L.M., Birren B., Nusbaum C., Zody M.C., Baldwin J., Devon K., Dewar K., Doyle M., FitzHugh W. (2001). Initial sequencing and analysis of the human genome. Nature.

[79] Zhao X.-Q., Bai F.-W. (2012). Zinc and yeast stress tolerance: Micronutrient plays a big role. J. Biotechnol..

[80] Burns K.H. (2017). Transposable elements in cancer. Nat. Rev. Cancer.

[81] Hartwig A. (2000). Recent advances in metal carcinogenicity. Pure Appl. Chem..

[82] Schrauzer G.N. (2021). The role of trace elements in the etiology of cancer. In Proceedings of the First International Workshop Neuherberg, Federal Republic of Germany, April 1980. https://www.degruyter.com/document/doi/10.1515/9783112417249-021/html.

[83] Heavy Metals in The Environment—Bibudhendra Sarkar—Google Książki. https://books.google.pl/books?id=OJboWGzbq1EC&pg=PA124&lpg=PA124&dq=P.L.+Goering,+M.P.+Waalkes,+C.D.+Klaassen,+in:+R.A.+Goyer,+M.G.+Cherian+(Eds.),+Handbook+of+Experimental+Pharmacology,+vol.+115,+Toxicology+of+Metals,+Biochemical+Effects,+Springer,+New+York,+1994,+pp.+189%E2%80%93214&source=bl&ots=lSx-SQ82q4&sig=ACfU3U2r7Tfm2d4p8mZAUE97Dzeg0ddNTg&hl=pl&sa=X&ved=2ahUKEwjVocb51r_6AhVmxosKHQlECIIQ6AF6BAgIEAM#v=onepage&q=P.L.%20Goering%2C%20M.P.%20Waalkes%2C%20C.D.%20Klaassen%2C%20in%3A%20R.A.%20Goyer%2C%20M.G.%20Cherian%20(Eds.)%2C%20Handbook%20of%20Experimental%20Pharmacology%2C%20vol.%20115%2C%20Toxicology%20of%20Metals%2C%20Biochemical%20Effects%2C%20Springer%2C%20New%20York%2C%201994%2C%20pp.%20189%E2%80%93214&f=false.

[84] Kägi J.H. (1991). Overview of metallothionein. Methods Enzymol..

[85] Waisberg M., Joseph P., Hale B., Beyersmann D. (2003). Molecular and cellular mechanisms of cadmium carcinogenesis. Toxicology.

[86] Elinder C.G., Kjellström T., Friberg L., Linnman B.L.L. (1976). Cadmium in kidney cortex, liver, and pancreas from Swedish autopsies. Estimation of biological half time in kidney cortex, considering calorie intake and smoking habits. Arch. Environ. Health Int. J..

[87] Yang J.-M., Arnush M., Chen Q.-Y., Wu X.-D., Pang B., Jiang X.-Z. (2003). Cadmium-induced damage to primary cultures of rat Leydig cells. Reprod. Toxicol..

[88] Price D.J., Joshi J.G. (1983). Ferritin. Binding of beryllium and other divalent metal ions. J. Biol. Chem..

[89] Waalkes M.P. (2000). Cadmium carcinogenesis in review. J. Inorg. Biochem..

[90] Verougstraete V., Lison D., Hotz P. (2003). Cadmium, lung and prostate cancer: A systematic review of recent epidemiological data. J. Toxicol. Environ. Health Part B.

[91] Şaplakoǧlu U., Işcan M. (1998). Sister chromatid exchanges in human lymphocytes treated in vitro with cadmium in G(o) and S phase of their cell cycles. Mutat. Res. Toxicol. Environ. Mutagen..

[92] Pearson C., Prozialeck W. (2001). E-Cadherin, beta-Catenin and cadmium carcinogenesis. Med. Hypotheses.

[93] Flora S.J.S., Mittal M., Mehta A. (2008). Heavy metal induced oxidative stress & its possible reversal by chelation therapy. Indian J. Med. Res..

[94] Flora S.J.S., Pachauri V. (2010). Chelation in metal intoxication. Int. J. Environ. Res. Public Health.

[95] Sartor F.A., Rondia D.J., Claeys F.D., Staessen J.A., Lauwerys R.R., Bernard A.M., Buchet J.P., Roels H.A., Bruaux P.J., Ducoffre G.M. (1992). Impact of environmental cadmium pollution on cadmium exposure and body burden. Arch. Environ. Health Int. J..

[96] Adams S.V., Passarelli M.N., Newcomb P.A. (2011). Cadmium exposure and cancer mortality in the Third National Health and Nutrition Examination Survey cohort. Occup. Environ. Med..

[97] Hall J.M., Couse J.F., Korach K.S. (2001). The Multifaceted Mechanisms of Estradiol and Estrogen Receptor Signaling. J. Biol. Chem..

[98] Byrne C., Divekar S.D., Storchan G.B., Parodi D.A., Martin M.B. (2013). Metals and breast cancer. J. Mammary Gland. Biol. Neoplasia.

[99] Bernstein L. (2002). Epidemiology of endocrine-related risk factors for breast cancer. J. Mammary Gland. Biol. Neoplasia.

[100] Safe S. (2003). Cadmium’s disguise dupes the estrogen receptor. Nat. Med..

[101] Darbre P.D. (2006). Metalloestrogens: An emerging class of inorganic xenoestrogens with potential to add to the oestrogenic burden of the human breast. J. Appl. Toxicol..

[102] Pettersson K., Gustafsson J. (2001). Role of Estrogen Receptor Beta in Estrogen Action. Annu. Rev. Physiol..

[103] Martin M.B., Reiter R., Pham T., Avellanet Y.R., Camara J., Lahm M., Pentecost E., Pratap K., Gilmore B.A., Divekar S. (2003). Estrogen-like activity of metals in MCF-7 breast cancer cells. Endocrinology.

[104] Johnson M.D., Kenney N., Stoica A., Hilakivi-Clarke L., Singh B., Chepko G., Clarke R., Sholler P.F., Lirio A., Foss C. (2003). Cadmium mimics the in vivo effects of estrogen in the uterus and mammary gland. Nat. Med..

[105] (2020). Cervix Uteri. https://gco.iarc.fr/today/data/factsheets/cancers/23-Cervix-uteri-fact-sheet.pdf.

[106] Cogliano V., Baan R., Straif K., Grosse Y., Secretan B., El Ghissassi F. (2005). Carcinogenicity of human papillomaviruses. Lancet Oncol..

[107] Gustafsson L., Pontén J., Zack M., Adami H.-O. (1997). International incidence rates of invasive cervical cancer after introduction of cytological screening. Cancer Causes Control.

[108] International Collaboration of Epidemiological Studies of Cervical Cancer (2005). Carcinoma of the cervix and tobacco smoking: Collaborative reanalysis of individual data on 13,541 women with carcinoma of the cervix and 23,017 women without carcinoma of the cervix from 23 epidemiological studies. Int. J. Cancer.

[109] International Collaboration of Epidemiological Studies of Cervical Cancer (2006). Cervical carcinoma and reproductive factors: Collaborative reanalysis of individual data on 16,563 women with cervical carcinoma and 33,542 women without cervical carcinoma from 25 epidemiological studies. Int. J. Cancer.

[110] Smith J.S., Green J., de Gonzalez A.B., Appleby P., Peto J., Plummer M., Franceschi S., Beral V. (2003). Cervical cancer and use of hormonal contraceptives: A systematic review. Lancet.

[111] Schiffman M., Castle P.E., Jeronimo J., Rodriguez A.C., Wacholder S. (2007). Human papillomavirus and cervical cancer. Lancet.

[112] Wadhwa S.K., Kazi T.G., Afridi H.I., Talpur F.N. (2015). Interaction between carcinogenic and anti-carcinogenic trace elements in the scalp hair samples of different types of Pakistani female cancer patients. Clin. Chim. Acta.

[113] Balasubramaniyan N., Subramanian S., Sekar N., Bhuvarahamurthy V., Govindasamy S. (1994). Involvement of plasma copper, zinc and cadmium in human carcinoma of uterine cervix. Med. Oncol..

[114] Zhang J., Nazeri S.A., Sohrabi A. (2021). Lead (Pb) exposure from outdoor air pollution: A potential risk factor for cervical intraepithelial neoplasia related to HPV genotypes. Environ. Sci. Pollut. Res..

[115] (2020). Corpus Uteri Source: Globocan 2020. https://gco.iarc.fr/today/data/factsheets/cancers/24-Corpus-uteri-fact-sheet.pdf.

[116] Hill H.A., Eley J.W., Harlan L.C., Greenberg R.S., Ii R.J.B., Chen V.W. (1996). Racial differences in endometrial cancer survival: The black/white cancer survival study. Obstet. Gynecol..

[117] Clement P.B., Young R.H. (2002). Endometrioid carcinoma of the uterine corpus: A review of its pathology with emphasis on recent advances and problematic aspects. Adv. Anat. Pathol..

[118] Neven P., De Muylder X., Van Belle Y., Van-Hooff I., Vanderick G. (1998). Longitudinal hysteroscopic follow-up during tamoxifen treatment. Lancet.

[119] Parazzini F., La Vecchia C., Negri E., Fedele L., Balotta F. (1991). Reproductive factors and risk of endometrial cancer. Am. J. Obstet. Gynecol..

[120] Van Gorp T., Neven P. (2002). Endometrial safety of hormone replacement therapy: Review of literature. Maturitas.

[121] Anderson G.L., Judd H.L., Kaunitz A.M., Barad D.H., Beresford S.A.A., Pettinger M., Liu J., McNeeley S.G., Lopez A.M., Women’s Health Initiative Investigators (2003). Effects of estrogen plus progestin on gynecologic cancers and associated diagnostic procedures: The Women’s Health Initiative randomized trial. JAMA.

[122] Beral V., Bull D., Reeves G., Million Women Study Collaborators (2005). Endometrial cancer and hormone-replacement therapy in the Million Women Study. Lancet.

[123] Hinkula M., Pukkala E., Kyyrönen P., Kauppila A. (2002). Grand multiparity and incidence of endometrial cancer: A population-based study in Finland. Int. J. Cancer.

[124] Deligeoroglou E., Michailidis E., Creatsas G. (2003). Oral contraceptives and reproductive system cancer. Ann. N. Y. Acad. Sci..

[125] Viswanathan A.N., Feskanich D., De Vivo I., Hunter D.J., Barbieri R.L., Rosner B., Colditz G., Hankinson S.E. (2005). Smmoking and the risk of endometrial cancer: Results from the Nurses’ Health Study. Int. J. Cancer.

[126] Lesko S.M., Rosenberg L., Kaufman D.W., Helmrich S.P., Miller D.R., Strom B., Schottenfeld D., Rosenshein N.B., Knapp R.C., Lewis J. (1985). Cigarette smoking and the risk of endometrial cancer. N. Engl. J. Med..

[127] Wynn R.M. (1989). The Human Endometrium. Biology of the Uterus.

[128] Eriksen K.T., Halkjær J., Sørensen M., Meliker J.R., McElroy J.A., Tjønneland A., Raaschou-Nielsen O. (2014). Dietary cadmium intake and risk of breast, endometrial and ovarian cancer in danish postmenopausal women: A prospective cohort study. PLoS ONE.

[129] Adams S.V., Quraishi S.M., Shafer M.M., Passarelli M.N., Freney E.P., Chlebowski R.T., Luo J., Meliker J.R., Mu L., Neuhouser M.L. (2014). Dietary cadmium exposure and risk of breast, endometrial, and ovarian cancer in the women’s health initiative. Environ. Health Perspect..

[130] Akesson A., Julin B., Wolk A. (2008). Long-term dietary cadmium intake and postmenopausal endometrial cancer incidence: A population-based prospective cohort study. Cancer Res..

[131] McElroy J.A., Kruse R.L., Guthrie J., Gangnon R.E., Robertson J.D. (2017). Cadmium exposure and endometrial cancer risk: A large midwestern U.S. population-based case-control study. PLoS ONE.

[132] Wieder-Huszla S., Chudecka-Głaz A., Cymbaluk-Płoska A., Karakiewicz B., Bosiacki M., Chlubek D., Jurczak A. (2022). Evaluation of the Concentration of Selected Elements in Patients with Cancer of the Reproductive Organs with Respect to Treatment Stage-Preliminary Study. Nutrients.

[133] Mittal K., Sebenik M., Irwin C., Yan Z., Popiolek D., Curtin J., Palazzo J. (2008). Preresence of endometrial adenocarcinoma in situ in complex atypical endometrial hyperplasia is associated with increased incidence of endometrial carcinoma in subsequent hysterectomy. Mod. Pathol..

[134] (2020). Ovary. https://gco.iarc.fr/today/data/factsheets/cancers/25-Ovary-fact-sheet.pdf.

[135] Torre L.A., Trabert B., DeSantis C.E., Miller K.D., Samimi G., Runowicz C.D., Gaudet M.M., Jemal A., Siegel R.L. (2018). Ovarian Cancer Statistics, 2018. CA Cancer J. Clin..

[136] Pietragalla A., Arcieri M., Marchetti C., Scambia G., Fagotti A. (2020). Ovarian cancer predisposition beyond BRCA1 and BRCA2 genes. Int. J. Gynecol. Cancer.

[137] La Vecchia C. (2017). Ovarian cancer: Epidemiology and risk factors. Eur. J. Cancer Prev..

[138] Julin B., Wolk A., Åkesson A. (2011). Dietary cadmium exposure and risk of epithelial ovarian cancer in a prospective cohort of Swedish women. Br. J. Cancer.

